# Single-step SNP-BLUP with on-the-fly imputed genotypes and residual polygenic effects

**DOI:** 10.1186/s12711-017-0310-9

**Published:** 2017-03-30

**Authors:** Matti Taskinen, Esa A. Mäntysaari, Ismo Strandén

**Affiliations:** Natural Resources Institute Finland (Luke), Myllytie 1, Jokioinen, Finland

## Abstract

**Background:**

Single-step genomic best linear unbiased prediction (BLUP) evaluation combines relationship information from pedigree and genomic marker data. The inclusion of the genomic information into mixed model equations requires the inverse of the combined relationship matrix ${\mathbf{H}}$, which has a dense matrix block for genotyped animals.

**Methods:**

To avoid inversion of dense matrices, single-step genomic BLUP can be transformed to single-step single nucleotide polymorphism BLUP (SNP-BLUP) which have observed and imputed marker coefficients. Simple block LDL type decompositions of the single-step relationship matrix ${\mathbf{H}}$ were derived to obtain different types of linearly equivalent single-step genomic mixed model equations with different sets of reparametrized random effects. For non-genotyped animals, the imputed marker coefficient terms in the single-step SNP-BLUP were calculated on-the-fly during the iterative solution using sparse matrix decompositions without storing the imputed genotypes. Residual polygenic effects were added to genotyped animals and transmitted to non-genotyped animals using relationship coefficients that are similar to imputed genotypes. The relationships were further orthogonalized to improve convergence of iterative methods.

**Results:**

All presented single-step SNP-BLUP models can be solved efficiently using iterative methods that rely on iteration on data and sparse matrix approaches. The efficiency, accuracy and iteration convergence of the derived mixed model equations were tested with a small dataset that included 73,579 animals of which 2885 were genotyped with 37,526 SNPs.

**Conclusions:**

Inversion of the large and dense genomic relationship matrix was avoided in single-step evaluation by using fully orthogonalized single-step SNP-BLUP formulations. The number of iterations until convergence was smaller in single-step SNP-BLUP formulations than in the original single-step GBLUP when heritability was low, but increased above that of the original single-step when heritability was high.

## Background

 The first model to simultaneously combine genomic information with non-genotyped animal information was single-step best linear unbiased prediction (BLUP) [1, 2] or ssGBLUP. When the number of genotyped animals is large, ssGBLUP may become computationally infeasible for practical purposes because it requires the inverses of dense matrices of size equal to the number of genotyped animals, particularly the inverse of the genomic relationship matrix ${{\mathbf{G}}_g^{-1}}$. In addition, matrix ${\mathbf{G}}_g$ can be singular when the number of genotyped individuals exceeds the number of markers. Computational challenges may have been a reason for the slow adoption of ssGBLUP instead of a multi-step approach. A computationally scalable alternative, the algorithm for proven and young (APY), has been suggested [3]. In APY, a sparse ${{\mathbf{G}}_{APY}^{-1}}$ approximation to the ${{\mathbf{G}}_g^{-1}}$ matrix is created by setting a diagonal matrix for a group of individuals. In practice, APY has been able to reduce computational costs significantly when the number of genotyped animals is very large [4, 5]. However, different sets of core animals in APY will give different evaluations, which may affect selection decisions.

An alternative formulation called hereinafter single-step single nucleotide polymorphism BLUP (ssSNP-BLUP) [6] overcomes some of the major computational challenges in ssGBLUP. In particular, there is no need to construct or invert the genomic relationship matrix ${\mathbf{G}}_g$. The original idea in ssSNP-BLUP circumvents the problems in ssGBLUP by predicting or imputing genotypes for non-genotyped animals, and relying on computationally less demanding SNP-based prediction instead of breeding value based prediction. An additional advantage is that the marker effect solutions are easier to use for interim predictions.

The ssSNP-BLUP has some computational challenges as well. A simple implementation for ssSNP-BLUP generates and stores genotypes of all SNPs for all animals. This will lead to very large disk storage and fast reading requirements that will prohibit use of the approach for large populations. An alternative is to make the required genotype imputations “on-the-fly” instead of storing the very large amount of imputed genotypes to file. For practical purposes, the on-the-fly imputation requires a fast computing approach for the imputation step and/or fast convergence of the iterative method.

The original description for ssSNP-BLUP approach presents a wider range of models than ssGBLUP such as the use of a number of different Bayesian SNP model formulations [6]. In ssGBLUP, in contrast to ssSNP-BLUP, it is typical to include residual polygenic (RPG) information to enhance genomic information by including pedigree-based relationships into the genomic relationship matrix. Thus, the genomic relationship matrix is usually “adjusted” with part of the pedigree relationship matrix either to supply more additive relationship information or simply to make the genomic relationship matrix invertible. In the original ssSNP-BLUP formulation, this information has not been included. The computational challenges of ssGBLUP has led to the introduction of several equivalent models (e.g., [7–9]). However, these alternative approaches have had poor convergence by iterative methods [7, 9]. One reason is that the covariance structures have a poorer condition number which is a ratio of the largest and smallest eigenvalues and is used to measure numerical stability [9]. Some of the alternative versions of ssGBLUP have had SNP effects. Because ssSNP-BLUP is equivalent to ssGBLUP and ssGBLUP has many equivalent forms, alternative formulations of mixed model equations (MME) can be derived for ssSNP-BLUP as well.

In this paper, simple block LDL type decompositions [10] of the ssGBLUP relationship matrix are derived to obtain several linearly equivalent MME. This allows derivation and testing of several equivalent ssSNP-BLUP MME that avoid making and storing the imputed genotypes. In this paper, an explicit imputation of genotypes is not needed but instead we apply sparse matrix decompositions to attain pedigree-based regressions of evaluations of genotyped animals on non-genotyped animals using sparse matrix decompositions. Accuracy and iteration convergence of the derived equivalent MME are tested on a small Nordic dairy cattle dataset.

## Methods

For any model, an infinite number of equivalent models exist. In the following, first we recall the concept of equivalent models and how equivalent models can be made by attaching covariance information to the model design matrix. As an example, equivalence of genomic BLUP (GBLUP) and SNP-BLUP is presented. Then, ssGBLUP is recalled, and its covariance structure formulated using LDL decomposition. Equivalent MME are derived where information from the genomic relationship matrix are attached to the model design matrix similarly as shown for GBLUP and SNP-BLUP, resulting in ssSNP-BLUP. Finally, MME having orthogonalized random effects or diagonal covariance structures are derived in order to improve the convergence in iterative methods. A small dataset is used to illustrate performance of the derived equivalent models.

### Linearly equivalent models

Two mixed linear effects models, i.e.

1 $$\begin{aligned} {\mathbf{y}}&= {\mathbf{X}}{\mathbf{b}}+ {\mathbf{Z}}{\mathbf{u}}+ {\mathbf{e}}, Var({\mathbf{u}}) = {\mathbf{G}}, Var({\mathbf{e}}) = {\mathbf{R}}\end{aligned}$$

2 $$\begin{aligned} {\mathbf{y}}&= \widetilde{{\mathbf{X}}}\widetilde{{\mathbf{b}}}+ \widetilde{{\mathbf{Z}}}\widetilde{{\mathbf{u}}}+ \widetilde{{\mathbf{e}}}, Var(\widetilde{{\mathbf{u}}}) = \widetilde{{\mathbf{G}}}, Var(\widetilde{{\mathbf{e}}}) = \widetilde{{\mathbf{R}}} \end{aligned}$$

with the same observations ${\mathbf{y}}$ but different fixed (${\mathbf{b}}$ and $\widetilde{{\mathbf{b}}}$) and random effects (${\mathbf{u}}$ and $\widetilde{{\mathbf{u}}}$), residual errors (${\mathbf{e}}$ and $\widetilde{{\mathbf{e}}}$), model matrices (${\mathbf{X}}$, ${\mathbf{Z}}$, $\widetilde{{\mathbf{X}}}$, and $\widetilde{{\mathbf{Z}}}$) and variance structures (${\mathbf{G}}$, ${\mathbf{R}}$, $\widetilde{{\mathbf{G}}}$, and $\widetilde{{\mathbf{R}}}$), are said to be *linearly equivalent models* [11–13] if the expected values and the variances of the observations are equal. Thus, models (1) and (2) are equivalent if:

3 $$\begin{aligned} \left\{ \begin{array}{ll} &{}{\mathbf{X}}{\mathbf{b}}= \widetilde{{\mathbf{X}}}\widetilde{{\mathbf{b}}},\\ &{}{\mathbf{V}}= {\mathbf{Z}}{\mathbf{G}}{\mathbf{Z}}^{\prime} + {\mathbf{R}}= \widetilde{{\mathbf{Z}}}\widetilde{{\mathbf{G}}}\widetilde{{\mathbf{Z}}}^{\prime} + \widetilde{{\mathbf{R}}}= \widetilde{{\mathbf{V}}}. \end{array} \right. \end{aligned}$$

#### Separate equations for fixed and random effects

Mixed model (1) can be solved from separate equations for fixed and random effects [14] as:

4 $$\begin{aligned} \begin{aligned} \widehat{{\mathbf{b}}}&= {({\mathbf{X}}^{\prime}{{\mathbf{V}}}^{-1}{\mathbf{X}})}^{-1}{\mathbf{X}}^{\prime}{{\mathbf{V}}}^{-1}{\mathbf{y}}\\ \widehat{{\mathbf{u}}}&= {\mathbf{G}}{\mathbf{Z}}^{\prime}{{\mathbf{V}}}^{-1}({\mathbf{y}}- {\mathbf{X}}\widehat{{\mathbf{b}}}), \end{aligned} \end{aligned}$$

where matrix ${\mathbf{V}}$ needs to be invertible. The size of matrix ${\mathbf{V}}$ is the number of observations, which can be very large, and, therefore, solving the mixed model using this method is seldom feasible in practice.

#### Mixed model equations

In practice, Eq. (4) can be solved by Henderson’s MME [14] as follows:

5 $$\begin{aligned} \begin{bmatrix} {\mathbf{X}}^{\prime}{} {\mathbf{R}}^{-1}{} {\mathbf{X}}&\quad {\mathbf{X}}^{\prime}{} {\mathbf{R}}^{-1}{} {\mathbf{Z}}\\ {\mathbf{Z}}^{\prime}{} {\mathbf{R}}^{-1}{} {\mathbf{X}}&\quad {\mathbf{Z}}^{\prime}{} {\mathbf{R}}^{-1}{} {\mathbf{Z}}+{\mathbf{G}}^{-1}\\ \end{bmatrix} \begin{bmatrix} \widehat{{\mathbf{b}}}\\ \widehat{{\mathbf{u}}}\\ \end{bmatrix}= \begin{bmatrix} {\mathbf{X}}^{\prime}{} {\mathbf{R}}^{-1}{} {\mathbf{y}}\\ {\mathbf{Z}}^{\prime}{} {\mathbf{R}}^{-1}{} {\mathbf{y}}\\ \end{bmatrix}, \end{aligned}$$

where the variance matrix of the random effects ${\mathbf{G}}$ and the residual variance matrix ${\mathbf{R}}$ need to be invertible. MME (5) usually lead to more sparse matrix systems than Eq. (4).

#### Equivalent models by splitting the variance matrix

Suppose the variance matrix ${\mathbf{G}}$ can be expressed as a matrix product as follows:

6 $$\begin{aligned} {\mathbf{G}}= {\mathbf{M}}\widetilde{{\mathbf{G}}}{\mathbf{M}}^{\prime}, \end{aligned}$$

where ${\mathbf{M}}$ is rectangular and $\widetilde{{\mathbf{G}}}$ is an invertible square matrix. Here, the matrix $\widetilde{{\mathbf{G}}}$ could be, for example, an identity matrix and could have different dimensions than the ${\mathbf{G}}$ matrix.

Matrices ${\mathbf{G}}$ and ${\mathbf{Z}}$ are always together in Eq. (4). This allows us to re-parametrize the model as:

7 $$\begin{aligned} {\mathbf{V}}&= {\mathbf{Z}}{\mathbf{G}}{\mathbf{Z}}^{\prime} + {\mathbf{R}}\\&= {\mathbf{Z}}{\mathbf{M}}\widetilde{{\mathbf{G}}}{\mathbf{M}}^{\prime}{\mathbf{Z}}^{\prime} + {\mathbf{R}}\nonumber \\&= \widetilde{{\mathbf{Z}}}\widetilde{{\mathbf{G}}}\widetilde{{\mathbf{Z}}}^{\prime} + \widetilde{{\mathbf{R}}}\nonumber = \widetilde{{\mathbf{V}}}, \end{aligned}$$

and thereafter:

8 $$\begin{aligned} \widehat{{\mathbf{u}}}&= {\mathbf{M}}\widetilde{{\mathbf{G}}}{\mathbf{M}}^{\prime}{\mathbf{Z}}^{\prime}{{\mathbf{V}}}^{-1}({\mathbf{y}}{-}{\mathbf{X}}\widehat{{\mathbf{b}}}) \nonumber \\&= {\mathbf{M}}\widetilde{{\mathbf{G}}}\widetilde{{\mathbf{Z}}}^{\prime}{\widetilde{{\mathbf{V}}}}^{-1}({\mathbf{y}}{-}{\mathbf{X}}\widehat{{\mathbf{b}}}) = {\mathbf{M}}\widetilde{{\mathbf{u}}}, \end{aligned}$$

where the equivalent model has the same residual variance matrix ($\widetilde{{\mathbf{R}}}= {\mathbf{R}}$), the new model matrix $\widetilde{{\mathbf{Z}}}= {\mathbf{Z}}{\mathbf{M}}$, and the random effects $\widetilde{{\mathbf{u}}}$ are defined as:

9 $$\begin{aligned} \widetilde{{\mathbf{u}}}= \widetilde{{\mathbf{G}}}\widetilde{{\mathbf{Z}}}^{\prime}{\widetilde{{\mathbf{V}}}}^{-1}({\mathbf{y}}- {\mathbf{X}}\widehat{{\mathbf{b}}}). \end{aligned}$$

Now, according to Eq. (3), the quantities with a tilde together with the same observation vector ${\mathbf{y}}$, the original fixed effects ($\widetilde{{\mathbf{b}}}= \widehat{{\mathbf{b}}}$), and design matrix ($\widetilde{{\mathbf{X}}}= {\mathbf{X}}$) form a linearly equivalent model (2).

#### Linearly equivalent MME

Original fixed effects $\widehat{{\mathbf{b}}}$ and the new random effects $\widetilde{{\mathbf{u}}}$ of the linearly equivalent model can be solved, similarly as in Eq. (4), from:

10 $$\begin{aligned} \begin{aligned} \widehat{{\mathbf{b}}}&= {({\mathbf{X}}^{\prime}{\widetilde{{\mathbf{V}}}}^{-1}{\mathbf{X}})}^{-1}{\mathbf{X}}^{\prime}{\widetilde{{\mathbf{V}}}}^{-1}{\mathbf{y}}\\ \widetilde{{\mathbf{u}}}&= \widetilde{{\mathbf{G}}}\widetilde{{\mathbf{Z}}}^{\prime}{\widetilde{{\mathbf{V}}}}^{-1}({\mathbf{y}}- {\mathbf{X}}\widehat{{\mathbf{b}}}), \end{aligned} \end{aligned}$$

or from the corresponding MME (5):

11 $$\begin{aligned} \begin{bmatrix} {\mathbf{X}}^{\prime}{} {\mathbf{R}}^{-1}{} {\mathbf{X}}&\quad {\mathbf{X}}^{\prime}{} {\mathbf{R}}^{-1}\widetilde{{\mathbf{Z}}}\\ {\widetilde{{\mathbf{Z}}}}^{\prime}{} {\mathbf{R}}^{-1}{} {\mathbf{X}}&\quad {\widetilde{{\mathbf{Z}}}}^{\prime}{} {\mathbf{R}}^{-1}\widetilde{{\mathbf{Z}}}+\widetilde{{\mathbf{G}}}^{-1}\\ \end{bmatrix} \begin{bmatrix} \widehat{{\mathbf{b}}}\\ {\widetilde{{\mathbf{u}}}} \end{bmatrix}= \begin{bmatrix} {\mathbf{X}}^{\prime}{} {\mathbf{R}}^{-1}{} {\mathbf{y}}\\ {\widetilde{{\mathbf{Z}}}}^{\prime}{} {\mathbf{R}}^{-1}{} {\mathbf{y}}\\ \end{bmatrix}. \end{aligned}$$

Because of the equivalence $\widehat{{\mathbf{u}}}= {\mathbf{M}}\widetilde{{\mathbf{u}}}$ in Eq. (8), the original random effects $\widehat{{\mathbf{u}}}$ can be obtained from the solution of the *linearly equivalent MME* (11) by pre-multiplying the new random effects $\widetilde{{\mathbf{u}}}$ with matrix ${\mathbf{M}}$, i.e.

12 $$\begin{aligned} \begin{bmatrix} \widehat{{\mathbf{b}}}\\ \widehat{{\mathbf{u}}}\end{bmatrix}= \begin{bmatrix} {\mathbf{I}}_b&\quad 0\\ 0&\quad {\mathbf{M}}\\ \end{bmatrix} \begin{bmatrix} {\mathbf{X}}^{\prime}{} {\mathbf{R}}^{-1}{} {\mathbf{X}}&\quad {\mathbf{X}}^{\prime}{} {\mathbf{R}}^{-1}{\widetilde{{\mathbf{X}}}}\\ {\widetilde{{\mathbf{Z}}}}^{\prime}{} {\mathbf{R}}^{-1}{} {\mathbf{X}}&\quad {\widetilde{{\mathbf{Z}}}}^{\prime}{} {\mathbf{R}}^{-1}{\widetilde{{\mathbf{Z}}}}+\widetilde{{\mathbf{G}}}^{-1} \end{bmatrix}^{-1} \begin{bmatrix} {\mathbf{X}}^{\prime}{} {\mathbf{R}}^{-1}{} {\mathbf{y}}\\ {\widetilde{{\mathbf{Z}}}}^{\prime}{} {\mathbf{R}}^{-1}{} {\mathbf{y}}\\ \end{bmatrix}, \end{aligned}$$

where identity matrix ${\mathbf{I}}_b$ has dimension of $\widehat{{\mathbf{b}}}$. Note that the inverse of the MME matrix in Eq. (12) is usually not evaluated explicitly, but rather, the corresponding linear matrix equation is solved using either direct or iterative solution methods.

The original MME (5) can, thus, be solved from a modified linearly equivalent MME (11) where the number of random effects in $\widetilde{{\mathbf{u}}}$ could be smaller or larger than in $\widehat{{\mathbf{u}}}$, the variance structure $\widetilde{{\mathbf{G}}}$ could be easier to obtain or invert than the original ${\mathbf{G}}$, or the new matrix system could be otherwise numerically more efficient.

#### Presentation for GBLUP and SNP-BLUP

As an example, consider single-trait GBLUP MME [15]. The variance matrix of random effects ${\mathbf{u}}$ is based on a genomic relationship matrix (${\mathbf{G}}_g$), which describes the genomic relationships between individuals, i.e. $Var({\mathbf{u}}) = \sigma _u^2{\mathbf{G}}_g$. The genomic relationship matrix is usually fully dense and increases in order as the number of genotyped animals increases. The inverted genomic relationship matrix ${{\mathbf{G}}_g}^{-1}$ is needed in the solution of the GBLUP MME:

13 $$\begin{aligned} \begin{bmatrix} \widehat{{\mathbf{b}}}\\ \widehat{{\mathbf{u}}}\end{bmatrix} = \begin{bmatrix} {\mathbf{X}}^{\prime}{} {\mathbf{X}}&\quad {\mathbf{X}}^{\prime}{} {\mathbf{Z}}\\ {\mathbf{Z}}^{\prime}{} {\mathbf{X}}&\quad {\mathbf{Z}}^{\prime}{} {\mathbf{Z}}+\lambda {\mathbf{G}}^{-1}_g \end{bmatrix}^{-1} \begin{bmatrix} {\mathbf{X}}^{\prime}{} {\mathbf{y}}\\ {\mathbf{Z}}^{\prime}{} {\mathbf{y}}\\ \end{bmatrix}, \end{aligned}$$

where a single trait case is assumed for simplicity, ${\mathbf{R}}= \sigma _e^2{\mathbf{I}}$, and $\lambda = \frac{\sigma _e^2}{\sigma _u^2}$.

There are many ways to construct ${\mathbf{G}}_g$. Assume the genomic relationship matrix ${\mathbf{G}}_g$ can be expressed, in simplified form, using (centered and scaled) marker matrix ${\mathbf{Z}}_m$ [15] so that:

14 $$\begin{aligned} {\mathbf{G}}_g = {\mathbf{Z}}_m{\mathbf{Z}}_m^{\prime} = {\mathbf{M}}\widetilde{{\mathbf{G}}}{\mathbf{M}}^{\prime}, \end{aligned}$$

where

15 $$\begin{aligned} {\mathbf{M}}= {\mathbf{Z}}_m\;\;\; \text {and} \;\;\; \widetilde{{\mathbf{G}}}= {\mathbf{I}}_m, \end{aligned}$$

and ${\mathbf{I}}_m$ is an identity matrix of size equal to the number of markers. Now, a linearly equivalent MME (11), alternative to GBLUP MME (13), can be derived and original effects solved similarly as in (12) so that:

$$\begin{aligned} \begin{bmatrix} \widehat{{\mathbf{b}}}\\ \widehat{{\mathbf{u}}}\end{bmatrix}= \begin{bmatrix} {\mathbf{I}}_b& 0\\ 0&\quad {\mathbf{Z}}_m\\ \end{bmatrix} \begin{bmatrix} {\mathbf{X}}^{\prime}{} {\mathbf{X}}&\quad {\mathbf{X}}^{\prime}\widetilde{{\mathbf{Z}}}\\ {\widetilde{{\mathbf{Z}}}}^{\prime}{} {\mathbf{X}}&\quad {\widetilde{{\mathbf{Z}}}}^{\prime}\widetilde{{\mathbf{Z}}}+\lambda {\mathbf{I}}_m \end{bmatrix}^{-1} \begin{bmatrix} {\mathbf{X}}^{\prime}{} {\mathbf{y}}\\ {\widetilde{{\mathbf{Z}}}}^{\prime}{} {\mathbf{y}}\\ \end{bmatrix}, \end{aligned}$$

where $\widetilde{{\mathbf{Z}}}= {\mathbf{Z}}{\mathbf{Z}}_m$ and $\lambda$ is the same variance ratio as in Eq. (13). This equivalent MME system, known as the SNP-BLUP [16], has markers as random effects instead of individuals. Random effects are also “orthogonalized”, and, thus, inversion of the dense genomic relationship matrix ${\mathbf{G}}_g$ is avoided.

Note that, if the marker effects have unequal variances, the relationship matrix can be build as:

16 $$\begin{aligned} {\mathbf{G}}_g = {\mathbf{Z}}_m\mathbf{B}{\mathbf{Z}}_m^{\prime} = {\mathbf{M}}\widetilde{{\mathbf{G}}}{\mathbf{M}}^{\prime}, \end{aligned}$$

where

17 $$\begin{aligned} {\mathbf{M}}= {\mathbf{Z}}_m\;\;\; \text {and} \;\;\; \widetilde{{\mathbf{G}}}= \mathbf{B}, \end{aligned}$$

and the matrix $\mathbf{B}$ is a diagonal covariance matrix describing the variances of different marker effects. Now the solution of SNP-BLUP becomes:

$$\begin{aligned} \begin{bmatrix} \widehat{{\mathbf{b}}}\\ \widehat{{\mathbf{u}}}\end{bmatrix}= \begin{bmatrix} {\mathbf{I}}_b&\quad 0\\ 0&\quad {\mathbf{Z}}_m\\ \end{bmatrix} \begin{bmatrix} {\mathbf{X}}^{\prime}{} {\mathbf{X}}&\quad {\mathbf{X}}^{\prime}\widetilde{{\mathbf{Z}}}\\ {\widetilde{{\mathbf{Z}}}}^{\prime}{} {\mathbf{X}}&\quad {\widetilde{{\mathbf{Z}}}}^{\prime}\widetilde{{\mathbf{Z}}}+\lambda \mathbf{B}^{-1} \end{bmatrix}^{-1} \begin{bmatrix} {\mathbf{X}}^{\prime}{} {\mathbf{y}}\\ {\widetilde{{\mathbf{Z}}}}^{\prime}{} {\mathbf{y}} \end{bmatrix}. \end{aligned}$$

### Single-step SNP-BLUP

In ssGBLUP [1, 2], some individuals have genomic information while some have only pedigree information. The model for ssGBLUP is a special case of mixed effect models, where the between-animal relationships are modeled via the aggregated relationship matrix ${\mathbf{H}}$ [1, 2]. The relationships in ${\mathbf{H}}$ are described by the pedigree-based relationship matrix ${\mathbf{A}}$, and the genomic relationship matrix among genotyped animals by ${\mathbf{G}}_g$. Relationships among non-genotyped individuals are constructed from the pedigree but modified according to the relationships among genotyped animals.

Assuming that the non-genotyped individuals are denoted with sub- and super-scripts 1 and the genotyped individuals by sub- and super-scripts 2, the pedigree relationship matrix and its inverse are the following:

18 $$\begin{aligned} {\mathbf{A}}= \begin{bmatrix} {\mathbf{A}}_{11}&\quad {\mathbf{A}}_{12}\\ {\mathbf{A}}_{21}&\quad {\mathbf{A}}_{22} \end{bmatrix} \;\;\; \text {and} \;\;\; {{\mathbf{A}}}^{-1} = \begin{bmatrix} {\mathbf{A}}^{11}&\quad {\mathbf{A}}^{12}\\ {\mathbf{A}}^{21}&\quad {\mathbf{A}}^{22} \end{bmatrix}. \end{aligned}$$

Assume, as before, that the genomic relationship matrix has the form ${\mathbf{G}}_g = {\mathbf{Z}}_m{\mathbf{Z}}_m^{\prime}$. If the genotyped population contains identical twins, i.e. clones, or if there are more individuals than markers, the genomic relationship matrix ${\mathbf{G}}_g$ becomes singular and the usual MME (5) cannot be constructed. Hence, ${\mathbf{G}}_g$ is commonly adjusted by regressing it towards the pedigree relationship matrix ${\mathbf{A}}_{22}$ with:

19 $$\begin{aligned} {\mathbf{G}}_w = w{\mathbf{A}}_{22}+ (1-w){\mathbf{G}}_g, \end{aligned}$$

where $w$ is a scalar weight between 0 and 1 and can be interpreted as the relative weight on the polygenic effect [2, 15].

#### Single-step relationship matrix

The inverse of the ssGBLUP variance matrix ${\mathbf{H}}$ is [1, 2]:

20 $$\begin{aligned} {{\mathbf{H}}}^{-1}&= {\mathbf{A}}^{-1}{+} \begin{bmatrix} {\mathbf{0}}&\quad {\mathbf{0}}\\ {\mathbf{0}}&\quad {{\mathbf{G}}_w}^{-1}{-}{({\mathbf{A}}_{22})}^{-1} \end{bmatrix} \end{aligned}$$

21 $$= \begin{bmatrix} {{\mathbf{A}}}^{11}&\quad {{\mathbf{A}}}^{12}\\ {{\mathbf{A}}}^{21}&\quad {{\mathbf{A}}}^{22} + {{\mathbf{G}}_w}^{-1} - {{({\mathbf{A}}}_{22})}^{-1} \end{bmatrix}.$$

Using block LDL decomposition, it is equal to:

22 $$\begin{aligned} {{\mathbf{H}}}^{-1} = \begin{bmatrix} {\mathbf{I}}_1&\quad {\mathbf{0}}\\ -{\mathbf{A}}_{imp}^{\prime}&\quad {\mathbf{I}}_2 \end{bmatrix} \begin{bmatrix} {\mathbf{A}}^{11}&\quad {\mathbf{0}}\\ {\mathbf{0}}&\quad {{\mathbf{G}}_w}^{-1} \end{bmatrix} \begin{bmatrix} {\mathbf{I}}_1&\quad -{\mathbf{A}}_{imp}\\ {\mathbf{0}}&\quad {\mathbf{I}}_2 \end{bmatrix}, \end{aligned}$$

where ${\mathbf{I}}_1$ and ${\mathbf{I}}_2$ are identity matrices of size equal to the number of non-genotyped and genotyped animals, respectively. For imputation of genotypes:

23 $$\begin{aligned} {\mathbf{A}}_{imp} = {\mathbf{A}}_{12}{({\mathbf{A}}_{22})}^{-1} = {-}{({\mathbf{A}}^{11})}^{-1}{\mathbf{A}}^{12} \end{aligned}$$

is a regression prediction or an *imputation operator* that expands the genomic relationship information from the genotyped to the non-genotyped individuals [2, 6, 17]. By inverting the LDL decomposition of Eq. (22), the variance matrix ${\mathbf{H}}$ has a similar decomposition:

24 $$\begin{aligned} {\mathbf{H}}= \begin{bmatrix} {\mathbf{I}}_1& {\mathbf{A}}_{imp}\\ {\mathbf{0}}&\quad {\mathbf{I}}_2 \end{bmatrix} \begin{bmatrix} {({\mathbf{A}}^{11})}^{-1}& {\mathbf{0}}\\ {\mathbf{0}}& {\mathbf{G}}_w \end{bmatrix} \begin{bmatrix} {\mathbf{I}}_1& {\mathbf{0}}\\ {\mathbf{A}}_{imp}^{\prime}& {\mathbf{I}}_2 \end{bmatrix}. \end{aligned}$$

By using block matrix inversion identities (23) and

25 $$\begin{aligned} {({\mathbf{A}}^{11})}^{-1}&= {\mathbf{A}}_{11} - {\mathbf{A}}_{12}{({\mathbf{A}}_{22})}^{-1}{\mathbf{A}}_{21} \end{aligned}$$

to the “imputed” ${\mathbf{A}}_{22}$ matrix of ${\mathbf{G}}_w$ (19)

26 $$\begin{aligned} \begin{bmatrix} {\mathbf{A}}_{imp}\\ {\mathbf{I}}_2 \end{bmatrix} {\mathbf{A}}_{22} \begin{bmatrix} {\mathbf{A}}_{imp}^{\prime}&\quad {\mathbf{I}}_2 \end{bmatrix} = {\mathbf{A}}- \begin{bmatrix} {({\mathbf{A}}^{11})}^{-1}&\quad {\mathbf{0}}\\ {\mathbf{0}}&\quad {\mathbf{0}}\end{bmatrix}, \end{aligned}$$

the variance matrix ${\mathbf{H}}$ (24) can be alternatively expressed as:

27 $$\begin{aligned} {\mathbf{H}}= (1-w) \begin{bmatrix} {({\mathbf{A}}^{11})}^{-1}& {\mathbf{0}}\\ {\mathbf{0}}& {\mathbf{0}}\end{bmatrix} + w{\mathbf{A}}+ (1-w){\mathbf{G}}_{imp}, \end{aligned}$$

where ${\mathbf{G}}_{imp}$ is an imputed genomic relationship matrix as follows:

28 $$\begin{aligned} {\mathbf{G}}_{imp} = \begin{bmatrix} {\mathbf{A}}_{imp}\\ {\mathbf{I}}_2 \end{bmatrix} {\mathbf{G}}_g \begin{bmatrix} {\mathbf{A}}_{imp}^{\prime}&\quad {\mathbf{I}}_2 \end{bmatrix}. \end{aligned}$$

Note that this operates on ${\mathbf{G}}_{g}$ instead of ${\mathbf{G}}_{w}$. Also, note that ${\mathbf{G}}_{imp}$ has a size equal to the number of all animals. Genotyped animals have observed marker data but non-genotyped animals have imputed marker data.

#### Equivalent single-step MME

In the following, the single-step relationship matrix ${\mathbf{H}}$ will be expressed as six different decompositions equivalent to Eq. (6):

29 $$\begin{aligned} {\mathbf{H}}= {\mathbf{M}}_i\widetilde{{\mathbf{G}}}_i{\mathbf{M}}_i^{\prime}, \;\;\; \text {i} = 1,\dots ,6, \end{aligned}$$

for different matrices ${\mathbf{M}}_i$ and $\widetilde{{\mathbf{G}}}_i$. All of these lead to a linearly equivalent ssGBLUP MME system of new sets of random effects with, potentially, different numerical properties. The linearly equivalent MME of these modified sets of random effects are similar to Eq. (11) and the original effects can be solved similarly as in Eq. (12):

$$\begin{aligned} \begin{bmatrix} \widehat{{\mathbf{b}}}\\ \widehat{{\mathbf{u}}}\\ \end{bmatrix}= \begin{bmatrix} {\mathbf{I}}_b& {\mathbf{0}}\\ {\mathbf{0}}& {\mathbf{M}}_i \end{bmatrix} \begin{bmatrix} {\mathbf{X}}^{\prime}{} {\mathbf{X}}&\quad {\mathbf{X}}^{\prime}{\widetilde{{\mathbf{Z}}}}_i\\ {\widetilde{{\mathbf{Z}}}}^{\prime}_i{\mathbf{X}}&\quad {\widetilde{{\mathbf{Z}}}}^{\prime}_i{\widetilde{{\mathbf{Z}}}}_i+\lambda {\widetilde{{\mathbf{G}}}}_i^{-1} \end{bmatrix}^{-1} \begin{bmatrix} {\mathbf{X}}^{\prime}{} {\mathbf{y}}\\ {\widetilde{{\mathbf{Z}}}}^{\prime}_i{\mathbf{y}}\\ \end{bmatrix}, \end{aligned}$$

where $\widetilde{{\mathbf{Z}}}_i = {\mathbf{Z}}{\mathbf{M}}_i$ and a single trait case is assumed.

Note that in these equivalent MME, square matrix $\widetilde{{\mathbf{G}}}_i$ represents the covariance structure for the reparametrized random effects, ${\mathbf{M}}_i$ has a row for each original random effect in ${\mathbf{u}}$ to change the model matrix ${\mathbf{Z}}$, and $\widetilde{{\mathbf{Z}}}_i$ is the redefined model matrix.

In order to derive linearly equivalent MME for multiple trait cases:

30 $$\begin{aligned} {\mathbf{G}}= {\mathbf{G}}_0 \otimes {\mathbf{H}}, \end{aligned}$$

where $\otimes$ is the Kronecker product, the genetic (co)variance matrix ${\mathbf{G}}_0$ of size number of traits is assumed to have a decomposition:

31 $$\begin{aligned} {\mathbf{G}}_0 = {\mathbf{M}}_0\widetilde{{\mathbf{G}}}_0{\mathbf{M}}_0^{\prime}. \end{aligned}$$

For example, a simple case would have ${\mathbf{M}}_0$ as identity matrix and $\widetilde{{\mathbf{G}}}_0$ as ${\mathbf{G}}_0$. Now the variance matrix ${\mathbf{G}}$ can be expressed using the decompositions of the single-step relationship matrix (29) similarly as in Eq. (6):

32 $$\begin{aligned} {\mathbf{G}}&= {\mathbf{G}}_0 \otimes {\mathbf{H}}= ({\mathbf{M}}_0\widetilde{{\mathbf{G}}}_0{\mathbf{M}}_0^{\prime}) \otimes ({\mathbf{M}}_i\widetilde{{\mathbf{G}}}_i{\mathbf{M}}_i^{\prime}) \end{aligned}$$

33 $$= ({{\mathbf{M}}}_0\otimes {{\mathbf{M}}}_i)(\widetilde{{{\mathbf{G}}}}_0\otimes \widetilde{{{\mathbf{G}}}}_i) ({{\mathbf{M}}}_0\otimes {{\mathbf{M}}}_i)^{\prime}$$

34 $$= {{\mathbf{M}}}\widetilde{{{\mathbf{G}}}}{{\mathbf{M}}}^{\prime},$$

where

35 $$\begin{aligned} {\mathbf{M}}= {\mathbf{M}}_0\otimes {\mathbf{M}}_i \;\; \text {and} \;\; \widetilde{{\mathbf{G}}}= \widetilde{{\mathbf{G}}}_0\otimes \widetilde{{\mathbf{G}}}_i. \end{aligned}$$

Effects can then be solved from linearly equivalent multiple trait MME (12):

$$\begin{aligned} \begin{bmatrix} \widehat{{\mathbf{b}}}\\ \widehat{{\mathbf{u}}}\\ \end{bmatrix}= \begin{bmatrix} {\mathbf{I}}_b&\quad {\mathbf{0}}\\ {\mathbf{0}}&\quad {\mathbf{M}}_0 \otimes {\mathbf{M}}_i \end{bmatrix} \begin{bmatrix} {\mathbf{X}}^{\prime}{} {\mathbf{R}}^{-1}{} {\mathbf{X}}&\quad {\mathbf{X}}^{\prime}{} {\mathbf{R}}^{-1}\widetilde{{\mathbf{Z}}}_i\\ {\widetilde{{\mathbf{Z}}}}^{\prime}_i{\mathbf{R}}^{-1}{} {\mathbf{X}}&\quad {\widetilde{{\mathbf{Z}}}}^{\prime}_i{\mathbf{R}}^{-1}\widetilde{{\mathbf{Z}}}_i+\widetilde{{\mathbf{G}}}_0^{-1}\otimes \widetilde{{\mathbf{G}}}_i^{-1} \end{bmatrix}^{-1} \begin{bmatrix} {\mathbf{X}}^{\prime}{} {\mathbf{R}}^{-1}{} {\mathbf{y}}\\ \widetilde{{\mathbf{Z}}}^{\prime}_i{\mathbf{R}}^{-1}{} {\mathbf{y}}\\ \end{bmatrix},\end{aligned}$$

where $$\widetilde{{\mathbf{Z}}}_i = {\mathbf{Z}}({\mathbf{M}}_0\otimes {\mathbf{M}}_i)$$.

##### *Basic equivalent ssGBLUP MME*

 The LDL decomposition (24) can be used directly to build the first linearly equivalent ssGBLUP MME of this paper using ${\mathbf{H}}= {\mathbf{M}}_1\widetilde{{\mathbf{G}}}_1{\mathbf{M}}_1^{\prime}$ where:

36 $$\begin{aligned} {\mathbf{M}}_1 = \begin{bmatrix} {\mathbf{I}}_1& {\mathbf{A}}_{imp}\\ {\mathbf{0}}&\quad {\mathbf{I}}_2 \end{bmatrix} \;\; \text {and} \;\; \widetilde{{\mathbf{G}}}_1 = \begin{bmatrix} {({\mathbf{A}}^{11})}^{-1}& {\mathbf{0}}\\ {\mathbf{0}}& {\mathbf{G}}_w \end{bmatrix}. \end{aligned}$$

From the modified relationship matrix $\widetilde{{\mathbf{G}}}_1$ (36), it can be seen that this *basic equivalent ssGBLUP* MME has random effects for non-genotyped animals with variances ${({\mathbf{A}}^{11})}^{-1}$ and for genotyped animals with variances of the adjusted genomic relationship matrix ${\mathbf{G}}_w$. The number of effects in the system is, thus, the same as in the original ssGBLUP.

##### *Basic RPG ssSNP-BLUP MME*

 Other linearly equivalent ssGBLUP MME of the form (29) can be derived as well. Note that the adjusted genomic relationship matrix ${\mathbf{G}}_w$ in Eq. (19) can be expressed by matrix products as follows:

37 $$\begin{aligned} {\mathbf{G}}_w&= \begin{bmatrix} {\mathbf{I}}_2& {\mathbf{I}}_2 \end{bmatrix} \begin{bmatrix} w{\mathbf{A}}_{22}&\quad {\mathbf{0}}\\ {\mathbf{0}}&\quad (1-w){\mathbf{G}}_g \end{bmatrix} \begin{bmatrix} {\mathbf{I}}_2\\ {\mathbf{I}}_2 \end{bmatrix} \end{aligned}$$

38 $$= \begin{bmatrix} {\mathbf{I}}_2& {\mathbf{Z}}_m \end{bmatrix} \begin{bmatrix} w{\mathbf{A}}_{22}& {\mathbf{0}}\\ {\mathbf{0}}& (1-w){\mathbf{I}}_m \end{bmatrix} \begin{bmatrix} {\mathbf{I}}_2\\ {\mathbf{Z}}_m^{\prime} \end{bmatrix},$$

where ${\mathbf{G}}_g = {\mathbf{Z}}_m{\mathbf{Z}}_m^{\prime}$. The second linearly equivalent ssGBLUP MME can be built by substituting ${\mathbf{G}}_w$ in Eq. (38) to Eq. (36):

39 $$\begin{aligned} \begin{aligned} {\mathbf{M}}_2&= \begin{bmatrix} {\mathbf{I}}_1&\quad \sqrt{w}{\mathbf{A}}_{imp}&\quad \sqrt{1-w}{\mathbf{A}}_{imp}{\mathbf{Z}}_m\\ {\mathbf{0}}&\quad \sqrt{w}{\mathbf{I}}_2&\quad \sqrt{1-w}{\mathbf{Z}}_m \end{bmatrix}\\ \widetilde{{\mathbf{G}}}_2&= \begin{bmatrix} {({\mathbf{A}}^{11})}^{-1}&\quad {\mathbf{0}}&\quad {\mathbf{0}}\\ {\mathbf{0}}&\quad {\mathbf{A}}_{22}&\quad {\mathbf{0}}\\ {\mathbf{0}}&\quad {\mathbf{0}}&\quad {\mathbf{I}}_m \end{bmatrix}. \end{aligned} \end{aligned}$$

In this form, we avoid the inverse of ${\mathbf{G}}_g$ in the MME (11). The coefficients $w$ and $(1-w)$ were also split using square roots to matrix ${\mathbf{M}}_2$ so that the new variance matrix $\widetilde{{\mathbf{G}}}_2$ can be inverted even when $w$ is 0 or 1. The first group of new random effects in Eq. (39), for the non-genotyped animals, is the same as in the first, basic equivalent ssGBLUP in Eq. (36). However, the genotyped animals now have random effects related through the variance matrix ${\mathbf{A}}_{22}$. Effects in this second effect group can be seen as *residual polygenic effects* that can describe effects that the marker effects are unable to model [8]. The third group of random effects are the marker effects as in SNP-BLUP (15) and so, this decomposition (39) can be called *basic RPG ssSNP-BLUP* MME. Compared to the original ssGBLUP, the second equivalent MME has marker effects in addition to the animal effects.

##### *Expanded RPG ssSNP-BLUP MME*

 A third linearly equivalent MME of form (29) can be derived from the alternative expression of matrix ${\mathbf{H}}$ in Eq. (27) and by splitting ${\mathbf{G}}_g = {\mathbf{Z}}_m{\mathbf{Z}}_m^{\prime}$ in Eq. (28) as:

40 $$\begin{aligned} {\mathbf{M}}_3&= \begin{bmatrix} \sqrt{1-w}{\mathbf{I}}_1&\quad \sqrt{w}{\mathbf{E}}_1&\quad \sqrt{1-w}{\mathbf{A}}_{imp}{\mathbf{Z}}_m\\ {\mathbf{0}}&\quad \sqrt{w}{\mathbf{E}}_2&\quad \sqrt{1-w}{\mathbf{Z}}_m \end{bmatrix}\\ \widetilde{{\mathbf{G}}}_3&= \begin{bmatrix} {({\mathbf{A}}^{11})}^{-1}&\quad {\mathbf{0}}&\quad {\mathbf{0}}\\ {\mathbf{0}}&\quad {\mathbf{A}}&\quad {\mathbf{0}}\\ {\mathbf{0}}&\quad {\mathbf{0}}&\quad {\mathbf{I}}_m \end{bmatrix}. \end{aligned}$$

Here, matrices ${\mathbf{E}}_1$ and ${\mathbf{E}}_2$ are rectangular sparse incidence matrices that select the subsets of non-genotyped and genotyped animals, respectively, from the ${\mathbf{A}}$ matrix. Both ${\mathbf{E}}_1$ and ${\mathbf{E}}_2$ have the same number of columns, i.e. number of all animals. Matrix ${\mathbf{E}}_1$ has a row for each non-genotyped and matrix ${\mathbf{E}}_2$ for each genotyped animal corresponding to animal’s column in matrices ${\mathbf{Z}}_1$ and ${\mathbf{Z}}_2$ of

41 $$\begin{aligned} {\mathbf{Z}}= \left[ {\mathbf{Z}}_1 \;\; {\mathbf{Z}}_2\right] . \end{aligned}$$

Each row of both ${\mathbf{E}}_1$ and ${\mathbf{E}}_2$ has only one non-zero element, a value one at the column corresponding to that animal’s location among all of the animals. Hence, when rows and columns of the matrices of all animals are in the same order as in matrix ${\mathbf{A}}$ in Eq. (18), matrix $$\begin{bmatrix} {\mathbf{E}}_1 \\ {\mathbf{E}}_2 \end{bmatrix}$$ is an identity matrix of the size of all animals.

The third equivalent MME (40) has three groups of effects similar to the second MME (39). The third effect group has, again, the orthogonal marker effects, and so this formulation is a ssSNP-BLUP as well. The first effect group, for the non-genotyped animals has, however, a constant multiplier $\sqrt{1-w}$. Also, the second group, related through the pedigree relationship matrix ${\mathbf{A}}$, has now effects for all animals, and not just for the genotyped animals. Thus, in this third *expanded RPG ssSNP-BLUP* MME, the non-genotyped animals have two sets of random effects.

##### *Special cases of equivalent ssGBLUP MME*

 These three equivalent MME, (36), (39) and (40), will approach the usual animal model when $w \rightarrow 1$. At the limit ($w = 1$), the expanded RPG ssSNP-BLUP MME 3 (40) has clearly the recognizable covariance structure of ${\mathbf{A}}$. The basic equivalent ssGBLUP MME 1 (36) and the basic RPG ssSNP-BLUP MME 2 (39) are models where the genotyped animals act as base animals and the non-genotyped animals are regressed on them.

For the other direction of $w \rightarrow 0$, the basic equivalent ssGBLUP MME 1 (36) converges to an alternative presentation of the standard ssGBLUP. However, it divides the breeding values of non-genotyped animals into regressions on genotyped animals and into non-imputed breeding values that are not conditional on them. Similarly, at the limit $w = 0$ the basic and the expanded RPG ssSNP-BLUP MME, 2 (39) and 3 (40), coincide with the simple ssSNP-BLUP without residual polygenic effects. For example, in the single trait case, the MME coefficient matrix of the basic RPG ssSNP-BLUP MME 2 (39) is

42 $$\begin{aligned} \begin{bmatrix} {\mathbf{X}}^{\prime}{\mathbf{X}}&\quad {\mathbf{X}}^{\prime}{\mathbf{Z}}_1&\quad {\mathbf{0}}&\quad {\mathbf{X}}^{\prime}{\mathbf{W}}\\ {\mathbf{Z}}_1^{\prime}{\mathbf{X}}&\quad {\mathbf{Z}}_1^{\prime}{\mathbf{Z}}_1 {+} \lambda {\mathbf{A}}^{11}&\quad {\mathbf{0}}&\quad {\mathbf{Z}}_1^{\prime}{\mathbf{W}}\\ {\mathbf{0}}&\quad {\mathbf{0}}&\quad \lambda {{\mathbf{A}}_{22}}^{-1}&\quad {\mathbf{0}}\\ {\mathbf{W}}^{\prime}{\mathbf{X}}&\quad {\mathbf{W}}^{\prime}{\mathbf{Z}}_1&\quad {\mathbf{0}}&\quad {\mathbf{W}}^{\prime}{\mathbf{W}}{+} \lambda {\mathbf{I}}_m \end{bmatrix}, \end{aligned}$$

where $${\mathbf{W}}= ({\mathbf{Z}}_1{\mathbf{A}}_{imp} + {\mathbf{Z}}_2){\mathbf{Z}}_m$$.

In the case where $w = 0$, i.e. there is no adjustment of the genomic relationship matrix and, therefore, no residual polygenic effects, the basic and the expanded RPG ssSNP-BLUP MME, 2 (39) and 3 (40), are essentially the same MME as was derived by Fernando et al. [6]. The main differences are that they have moved the centering term of the marker matrix ${\mathbf{Z}}_m$ into an additional fixed effect, and they proposed to solve the MME using Bayesian regression.

### Efficient implementation

The three linearly equivalent MME based on Eqs. (36), (39), and (40) contain inverted and non-inverted terms of the pedigree relationship matrix ${\mathbf{A}}$. In an efficient set up to solve these MME, all these terms can be expressed, or modified into a form that can be expressed, with sparse matrices or sparse decompositions of sparse matrices. This is expected to give three efficient implementations of these MME. In practice, matrix equations of these MME are assumed to be solved iteratively by the preconditioned conjugate gradient (PCG) algorithm. Then, only a matrix-vector product of the MME coefficient matrix times a vector is performed once every iteration.

#### Sparse matrices and decompositions

The inverse of the pedigree relationship matrix ${\mathbf{A}}^{-1}$ can be expressed efficiently [18] as:

43 $$\begin{aligned} {\mathbf{A}}^{-1} = {\mathbf{Q}}\left( {\mathbf{I}}-\tfrac{1}{2}\mathbf{P}\right) ^{\prime} \mathbf{D}\left({\mathbf{I}}-\tfrac{1}{2}\mathbf{P}\right){\mathbf{Q}}^{\prime} = {\mathbf{Q}}{\mathbf{L}}{\mathbf{L}}^{\prime}{\mathbf{Q}}^{\prime}, \end{aligned}$$

where animals are sorted in (reversed) age order from the youngest to the oldest using sparse permutation matrix ${\mathbf{Q}}^{\prime}$, so that matrix ${\mathbf{L}}= ({\mathbf{I}}-\frac{1}{2}\mathbf{P})^{\prime}\mathbf{D}^\frac{1}{2}$ becomes a lower triangular matrix in ${\mathbf{Q}}^{\prime}{\mathbf{A}}^{-1}{\mathbf{Q}}= {\mathbf{L}}{\mathbf{L}}^{\prime}$. The diagonal matrix $\mathbf{D}$ has values $4/(4-k-F_s)$ where $k$ is the number of known parents and $F_s$ is the sum of parent inbreeding coefficients. In the “parental matrix” $\mathbf{P}$ on row $i$, there are 1s in columns corresponding to parents of animal $i$. The parental matrix can be interpreted, together with identity matrix ${\mathbf{I}}$, as a very sparse lower triangular “Cholesky” matrix (${\mathbf{L}}$) [18].

The pedigree relationship matrix ${\mathbf{A}}$ can be expressed as the inverse of its inverse,

44 $$\begin{aligned} {\mathbf{A}}= {({\mathbf{A}}^{-1})}^{-1} = {\mathbf{Q}}{({\mathbf{L}}^{\prime})}^{-1}{{\mathbf{L}}}^{-1}{\mathbf{Q}}^{\prime}, \end{aligned}$$

and so the submatrices of the pedigree relationship matrix and its inverse (18) can be obtained by selecting the appropriate rows and columns as follows:

45 $$\begin{aligned} {\mathbf{A}}_{ij}= {\mathbf{E}}_i{\mathbf{Q}}{({\mathbf{L}}^{\prime})}^{-1}{{\mathbf{L}}}^{-1}{\mathbf{Q}}^{\prime}{\mathbf{E}}_j^{\prime} \end{aligned}$$

46 $$\begin{aligned} {\mathbf{A}}^{ij}= {\mathbf{E}}_i{\mathbf{Q}}{\mathbf{L}}{\mathbf{L}}^{\prime}{\mathbf{Q}}^{\prime}{\mathbf{E}}_j^{\prime}, \end{aligned}$$

where $i,j = 1,2$. Matrix-vector products ${\mathbf{A}}^{ij} {\mathbf{x}}$ and ${\mathbf{A}}_{ij} {\mathbf{x}}$ can be efficiently computed using these decompositions [19, 20]. The submatrices ${\mathbf{A}}^{ij}$ are very sparse, so they could alternatively be expressed as separate sparse matrices.

The inverse of ${\mathbf{A}}^{-1}$ or particular parts of it (e.g. ${({\mathbf{A}}^{11})}^{-1}$) are, however, in general non-sparse and, thus, these inverse matrix terms should never be computed explicitly. The sparse submatrix ${\mathbf{A}}^{11}$ can be expressed using *sparsity preserving Cholesky factorization* so that:

47 $$\begin{aligned} {\mathbf{A}}^{11} = {\mathbf{Q}}_1{\mathbf{L}}_1{\mathbf{L}}_1^{\prime}{\mathbf{Q}}_1^{\prime}, \end{aligned}$$

where matrix ${\mathbf{L}}_1$ is sparse lower triangular and ${\mathbf{Q}}_1$ sparse permutation matrix. Note that matrix ${\mathbf{L}}_1$ has to be computed explicitly, as opposed to matrix ${\mathbf{L}}$ in Eqs. (43) to (46). Matrix ${\mathbf{L}}_1$ has some more fill-ins compared to ${\mathbf{A}}^{11}$ but is still very sparse and efficient in use. In [4], it was demonstrated that the computations remain affordable even when the dataset size grows.

Computations involving matrix inversions of ${\mathbf{A}}^{-1}$ or parts of it can be transformed into solutions of sparse matrix equation systems [20]:

48 $$\begin{aligned} {\mathbf{A}}{\mathbf{v}} &= {\mathbf{Q}}{({\mathbf{L}}^{\prime})}^{-1}{{\mathbf{L}}}^{-1}{\mathbf{Q}}^{\prime}{\mathbf{v}}\nonumber \\ &= {\mathbf{Q}}\left( {\mathbf{L}}^{\prime}{\backslash }\left( {\mathbf{L}}{\backslash }({\mathbf{Q}}^{\prime}{\mathbf{v}})\right) \right) \end{aligned}$$

49 $$\begin{aligned} {({\mathbf{A}}^{11})}^{-1}{\mathbf{v}}_1= & {} {\mathbf{Q}}_1\left( {\mathbf{L}}_1^{\prime}{\backslash }\left( {\mathbf{L}}_1{\backslash }({\mathbf{Q}}_1^{\prime}{\mathbf{v}}_1)\right) \right) , \end{aligned}$$

where ${\mathbf{v}}$ and ${\mathbf{v}}_1$ are vectors of appropriate sizes into which the inverse matrix operations are performed. Here the backslash (${\backslash }$) is an operator indicating *forward or backward substitutions* and emphasizes the importance of avoiding inverting matrices. In other words, ${\mathbf{L}}{\backslash }{\mathbf{y}}$ is the solution ${\mathbf{x}}$ of equation ${\mathbf{L}}{\mathbf{x}}= {\mathbf{y}}$ or can be expressed as solving (${\mathbf{L}}$,${\mathbf{y}}$). Note that the matrix products are carefully nested with parenthesis so that only matrix-vector operations are performed.

Furthermore, following [4, 9] the inverse of the matrix ${\mathbf{A}}_{22}$, needed in the inversion of the second modified relationship matrix $\widetilde{{\mathbf{G}}}_2$ in Eq. (39), can be expressed efficiently using block matrix inversion identity similar to Eq. (25) as:

50 $$\begin{aligned} {({\mathbf{A}}_{22})}^{-1} = {\mathbf{A}}^{22} - {\mathbf{A}}^{21}{({\mathbf{A}}^{11})}^{-1}{\mathbf{A}}^{12}, \end{aligned}$$

where all terms can be computed using Eqs. (45) to (49).

#### On-the-fly imputation operation

The derived equivalent formulations in Eqs. (36), (39), and (40) contain imputation operator ${\mathbf{A}}_{imp} = -{({\mathbf{A}}^{11})}^{-1}{\mathbf{A}}^{12}$ (23) in matrices ${\mathbf{M}}_i$ that are needed when operating with the modified model matrix $\widetilde{{\mathbf{Z}}}_i = {\mathbf{Z}}{\mathbf{M}}_i$ and its transpose $\widetilde{{\mathbf{Z}}}^{\prime}_i = {\mathbf{M}}^{\prime}_i{\mathbf{Z}}^{\prime}$, and when calculating the original random effects in Eq. (12). When the MME are solved by the PCG iteration algorithm, the core of the algorithm is a multiplication of the so-called direction vector ${\mathbf{v}}$ by the left hand side of the MME (11). In this multiplication, the imputation operator, as part of the MME coefficient matrix, operates either with a part of the vector pertaining to random effects of the genotyped animals (i.e. ${\mathbf{A}}_{imp}{\mathbf{v}}_2$) or to a vector of the marker effects ${\mathbf{v}}_m$ through the marker matrix (i.e. ${\mathbf{A}}_{imp}{\mathbf{Z}}_m{\mathbf{v}}_m$). Thus, in the transpose $\widetilde{{\mathbf{Z}}}^{\prime}_i$ side, the imputation term operates on a vector of size equal to the number of non-genotyped animals (i.e. ${\mathbf{A}}_{imp}^{\prime}{\mathbf{v}}_1$).

In all cases, the size of the vector term that operates on ${({\mathbf{A}}^{11})}^{-1}$ equals the number of non-genotyped animals, i.e. size of ${\mathbf{v}}_1$. For example, the *imputed genomic marker data* term, $-{({\mathbf{A}}^{11})}^{-1}{\mathbf{A}}^{12}{\mathbf{Z}}_m{\mathbf{v}}_m$, that expands the genomic information from genotyped to non-genotyped animals, can be calculated using:

51 $$\begin{aligned} \widetilde{{\mathbf{v}}}_2= {\mathbf{Z}}_m{\mathbf{v}}_m \end{aligned}$$

52 $$\begin{aligned} {\mathbf{v}}_1= {\mathbf{A}}^{12}\widetilde{{\mathbf{v}}}_2 \end{aligned}$$

and Eq. (49) so that:

53 $$\begin{aligned} -{({\mathbf{A}}^{11})}^{-1}{\mathbf{A}}^{12}{\mathbf{Z}}_m{\mathbf{v}}_m = -{\mathbf{Q}}_1\left( {\mathbf{L}}_1^{\prime}{\backslash }\left( {\mathbf{L}}_1{\backslash }({\mathbf{Q}}_1^{\prime}{\mathbf{v}}_1)\right) \right) . \end{aligned}$$

Vector ${\mathbf{v}}_1$ is calculated from Eq. (46), or without constructing any of the matrices by using rules for ${\mathbf{A}}^{-1}$ by pedigree information [18], or, alternatively, as a sparse matrix-vector product of separate sparse matrix ${\mathbf{A}}^{12}$.

Note that the actual imputation of the genomic marker information is not needed. The imputation operation is performed only implicitly, “on-the-fly” during the iterative solution without the need to use, for example, disk storage. In the normal imputation process [6], the marker information needs to be calculated for thousands, or even hundreds of thousands marker vectors of genotyped animals, i.e. columns of marker matrix ${\mathbf{Z}}_m$. The predicted marker data matrix contains real numbers and can be very large, and, thus, takes a lot of time and disk space to generate and use.

In the on-the-fly imputation process of genetic effects, however, imputation is an operation on a “projection vector” of the genotyped animals, i.e. a linear combination of the marker vectors. It needs to be performed only twice within each iteration round for each trait. Once for matrix $\widetilde{{\mathbf{Z}}}_i$ and another time for the transpose $\widetilde{{\mathbf{Z}}}^{\prime}_i$ in matrix multiplication of MME coefficient matrix of Eq. (11). The imputation operation is also needed once before the iteration when calculating the right-hand-side for the new random effects ($\widetilde{{\mathbf{Z}}}_i^{\prime}{{\mathbf{R}}}^{-1}{\mathbf{y}}$) in Eq. (11) and once at the end of the iteration in order to retrieve the original random effects ($\widehat{{\mathbf{u}}}= {\mathbf{M}}_i\widetilde{{\mathbf{u}}}$) in Eq. (12).

#### Orthogonalization of random effects

In the SNP-BLUP versions of the derived equivalent ssGBLUP, in Eqs. (39) and (40), the marker effects are orthogonal, i.e. their covariance matrix is diagonal. It turns out that the PCG iteration numbers of these two equivalent ssSNP-BLUP MME are considerably larger than the original ssGBLUP. In Eq. (39), the RPG effects and genomic values predicted by SNPs have colinearity, and in Eq. (40), the RPG and the animal effects for non-genotyped animals are difficult to separate.

The key for maintaining good numerical properties of the original ssGBLUP seems to be to “orthogonalize” the remaining new random effects, too. The remaining variance structures can be orthogonalized by splitting the variance matrices and attaching the two “halfs” into the coefficient matrices ${\mathbf{M}}$ as in Eqs. (6) and (29).

The term ${({\mathbf{A}}^{11})}^{-1}$ in Eqs. (39) and (40) can be orthogonalized by using the sparse Cholesky factorization in Eq. (47) as follows:

54 $$\begin{aligned} {({\mathbf{A}}^{11})}^{-1} = {\mathbf{M}}_{11}\widetilde{{\mathbf{G}}}_{11}{\mathbf{M}}_{11}^{\prime}, \end{aligned}$$

where

55 $$\begin{aligned} {\mathbf{M}}_{11} = {\mathbf{Q}}_1{({\mathbf{L}}_1^{\prime})}^{-1} \;\; \text {and} \;\; \widetilde{{\mathbf{G}}}_{11} = {\mathbf{I}}_1. \end{aligned}$$

Note that the permutation operator ${\mathbf{Q}}_1$ can be performed outside the inverse operator. However, the term ${\mathbf{A}}_{22}$ in Eq. (39) seems to be much more difficult to decompose. Still, it can be expressed using the sparse decomposition of the full matrix ${\mathbf{A}}$ in Eq. (44) as:

56 $$\begin{aligned} {\mathbf{A}}_{22} = {\mathbf{E}}_2{\mathbf{A}}{\mathbf{E}}_2^{\prime} = {\mathbf{M}}_{22}\widetilde{{\mathbf{G}}}_{22}{\mathbf{M}}_{22}^{\prime}, \end{aligned}$$

where

57 $$\begin{aligned} {\mathbf{M}}_{22} = \widetilde{{\mathbf{A}}}^\frac{1}{2}_2 \;\; \text {and} \;\; \widetilde{{\mathbf{G}}}_{22} = {\mathbf{I}}, \end{aligned}$$

and

58 $$\begin{aligned} \widetilde{{\mathbf{A}}}^\frac{1}{2}_k = {\mathbf{E}}_k{\mathbf{Q}}{({\mathbf{L}}^{\prime})}^{-1}, \;\; k = 1,2. \end{aligned}$$

The rectangular matrix $\widetilde{{\mathbf{A}}}^\frac{1}{2}_2$ has dimensions number of genotyped individuals times total number of individuals, hence the trade-off here is that Eq. (56) will expand the second random effect group of Eq. (39) from genotyped to all individuals (size of ${\mathbf{I}}$).

Using Eqs. (54), (56), and (58), the linearly equivalent MME (39) and (40) can be “orthogonalized” into fourth:

59 $$\begin{aligned} {\mathbf{M}}_4 = \begin{bmatrix} {\mathbf{M}}_{11}&\quad \sqrt{w}{\mathbf{A}}_{imp}\widetilde{{\mathbf{A}}}^\frac{1}{2}_2&\quad \sqrt{1-w}{\mathbf{A}}_{imp}{\mathbf{Z}}_m\\ {\mathbf{0}}&\quad \sqrt{w}\widetilde{{\mathbf{A}}}^\frac{1}{2}_2&\quad \sqrt{1-w}{\mathbf{Z}}_m \end{bmatrix} \end{aligned},$$

and fifth

60 $$\begin{aligned} {\mathbf{M}}_5 = \begin{bmatrix} \sqrt{1-w}{\mathbf{M}}_{11}&\quad \sqrt{w}\widetilde{{\mathbf{A}}}^\frac{1}{2}_1&\quad \sqrt{1-w}{\mathbf{A}}_{imp}{\mathbf{Z}}_m\\ {\mathbf{0}}&\quad \sqrt{w}\widetilde{{\mathbf{A}}}^\frac{1}{2}_2&\quad \sqrt{1-w}{\mathbf{Z}}_m \end{bmatrix} \end{aligned},$$

equivalent MME. Both of these linearly equivalent (29) ssSNP-BLUPs share the same orthogonal variance structure:

61 $$\begin{aligned} \widetilde{{\mathbf{G}}}_4 = \widetilde{{\mathbf{G}}}_5 = \begin{bmatrix} {\mathbf{I}}_1&\quad {\mathbf{0}}&\quad {\mathbf{0}}\\ {\mathbf{0}}&\quad {\mathbf{I}}&\quad {\mathbf{0}}\\ {\mathbf{0}}&\quad {\mathbf{0}}&\quad {\mathbf{I}}_m \end{bmatrix}, \end{aligned}$$

and, thus, both also have the same number of new random effects: random effects for the genotyped individuals, two sets of random effects for the non-genotyped individuals, and random effects for the markers. The difference in equivalent MME 4 (59) and 5 (60) is on how they divide the RPG on non-genotyped animals.

The fourth equivalent MME (59) can be called *orthogonal ssSNP-BLUP* MME, and the fifth MME (60), originating from the expanded RPG ssSNP-BLUP (40), *orthogonal expanded ssSNP-BLUP* MME.

#### Reduction of the number of effects by using ancestors of genotyped animals

Matrix ${\mathbf{A}}_{22}$, as the covariance structure for the genotyped animals, was reparametrized in Eq. (56) using the full pedigree relationship matrix ${\mathbf{A}}$. This reparametrization increases the number of corresponding new random effects from genotyped animals to all animals in the pedigree. However, computations involving ${\mathbf{A}}_{22}$ require only the genotyped individuals and their ancestors. Thus, to reduce the number of extra new effects, ${\mathbf{A}}_{22}$ can be expressed using a smaller pedigree and relationship matrix $\widehat{{\mathbf{A}}}$ containing the genotyped animals and their ancestors [4].

Let the inverse of the pedigree relationship matrix (43) of this smaller pedigree be:

62 $$\begin{aligned} {\widehat{{\mathbf{A}}}}^{-1} = \widehat{{\mathbf{Q}}}\widehat{{\mathbf{L}}}\widehat{{\mathbf{L}}}^{\prime}\widehat{{\mathbf{Q}}}^{\prime}, \end{aligned}$$

where $\widehat{{\mathbf{Q}}}$ and $\widehat{{\mathbf{L}}}$ are as before in Eq. (44) but involve genotyped animals and their ancestors only. Matrix ${\mathbf{A}}_{22}$ in Eq. (56) can then be represented using the smaller pedigree as:

63 $$\begin{aligned} {\mathbf{A}}_{22} = \widehat{{\mathbf{E}}}_2\widehat{{\mathbf{A}}}\widehat{{\mathbf{E}}}_2^{\prime} = \widehat{{\mathbf{M}}}_{22}\widehat{{\mathbf{G}}}_{22}\widehat{{\mathbf{M}}}_{22}^{\prime}, \end{aligned}$$

where

64 $$\begin{aligned} \widehat{{\mathbf{M}}}_{22} = \widehat{{\mathbf{E}}}_2\widehat{{\mathbf{Q}}}{(\widehat{{\mathbf{L}}}^{\prime})}^{-1} \;\; \text {and} \;\; \widehat{{\mathbf{G}}}_{22} = {\mathbf{I}}_{ganc}, \end{aligned}$$

$\widehat{{\mathbf{E}}}_2$ selects the genotyped individuals from the smaller pedigree, and the size of identity matrix ${\mathbf{I}}_{ganc}$ is the number of genotyped animals and their ancestors.

The sixth linearly equivalent, *reduced orthogonal ssSNP-BLUP* MME, can be derived from Eqs. (59) and (61) as:

65 $$\begin{aligned} \begin{aligned} {\mathbf{M}}_6&= \begin{bmatrix} {\mathbf{M}}_{11}&\quad \sqrt{w}{\mathbf{A}}_{imp}\widehat{{\mathbf{M}}}_{22}&\quad \sqrt{1-w}{\mathbf{A}}_{imp}{\mathbf{Z}}_m\\ {\mathbf{0}}&\quad \sqrt{w}\widehat{{\mathbf{M}}}_{22}&\quad \sqrt{1-w}{\mathbf{Z}}_m \end{bmatrix}\\ \widetilde{{\mathbf{G}}}_6&= \begin{bmatrix} {\mathbf{I}}_1&\quad {\mathbf{0}}&\quad {\mathbf{0}}\\ {\mathbf{0}}&\quad {\mathbf{I}}_{ganc}&\quad {\mathbf{0}}\\ {\mathbf{0}}&\quad {\mathbf{0}}&\quad {\mathbf{I}}_m \end{bmatrix}. \end{aligned} \end{aligned}$$

Here only the non-genotyped ancestors of the genotyped have two sets of random effects, all the other non-genotyped and all genotyped animals have single sets of effects, in addition to the marker effects.

### Data

The derived MME were tested using a small Nordic Red dairy cattle dataset and a simple model. The small dataset and the model were partially chosen in order to be able to use direct sparse matrix solutions of the original ssGBLUP to obtain accurate “correct solutions”. The data were deregressed proofs of milk yield that were based on estimated breeding values from the Nordic production trait evaluations by NAV (Nordic Evaluations, Denmark). There were 73,579 animals in the pedigree of which 2885 were genotyped. Genotyped animals together with their ancestors form a smaller pedigree of 6833 animals. The animals had been genotyped with the Illumina Bovine SNP50 Bead Chip (Illumina, San Diego, USA). The analysis used 37,526 SNPs that passed quality control. There were 66,426 non-genotyped and 1222 genotyped animals with phenotypes. Hence, 1663 animals had a genotype but no phenotype. We considered a single trait model and assumed a heritability of 0.5. The genomic data contained one pair of animals with identical genomic marker data and a couple of more near identical pairs that led to problems for the inversion of the genomic relationship matrix ${\mathbf{G}}_g$ without ${\mathbf{A}}_{22}$ adjustment, i.e. in the case $w = 0$.

### Comparison statistics

The original ssGBLUP with inverse variance matrix ${{\mathbf{H}}}^{-1}$ of Eq. (20) and the six linearly equivalent formulations of the form (29), from Eqs. (36), (39), (40), (59), (60), (61), and (65), were implemented and tested in an Octave [21] environment. Sparse matrix factorizations were based on CHOLMOD routines [22]. Six different weights $w$ were tested: 0.00, 0.01, 0.10, 0.20, 0.30, and 1.00. Because of the singularity in the inverse of the genomic relationship matrix ${{\mathbf{G}}_g}^{-1}$ with the test data, the original ssGBLUP matrix (20) and the first equivalent formulation (36) were not calculated when $w = 0$. The derived new formulations were compared against the original ssGBLUP, mainly focusing on efficiency, accuracy, and number of iterations.

Efficiency of the derived equivalent ssGBLUP formulations relies on the sparsity of the pedigree relationship matrices and their decompositions. Inverse matrix operations of these sparse matrices were transformed into solving sparse lower triangular matrix systems. The efficiency of these solving operations depend on the sparsity structure of the matrices, i.e. number of non-zero elements.

Accuracy of the formulations was tested by solving the MME with the PCG method using Octave’s PCG routine (pcg) with the diagonal of the MME coefficient matrix as the preconditioner, or without preconditioning. Convergence tolerance in pcg was relative residual norm. The tolerance was chosen to be small ($10^{-12}$) so that all solved effects, without doubt, converged. Accuracies, or rather the differences from the “exact solution”, were calculated as relative residual errors ($\widetilde{{\mathbf{e}}}_i$) between iteratively obtained MME solutions (${\mathbf{s}}_i$) and the direct solution (${\mathbf{s}}_{direct}$) of the original ssGBLUP:

66 $$\begin{aligned} \widetilde{{\mathbf{e}}}_i = \frac{\Vert {{\mathbf{s}}_{direct} - {\mathbf{s}}_i}\Vert }{\Vert {{\mathbf{s}}_{direct}}\Vert }, \end{aligned}$$

where subscript $i=1,\ldots ,6$ is the formulation number.

Implementations of the derived equivalent ssGBLUP formulation were not yet streamlined for speed and, thus, the execution times were neither optimal, nor comparable. The performance of the formulations is, therefore, tested by comparing the number of iterations of the iterative solution. The purpose was to demonstrate that the iteration counts are comparable to those obtained by the original ssGBLUP. With a larger number of genotyped individuals, the inversion of the genomic relationship matrix in the original ssGBLUP becomes a bottleneck and ssSNP-BLUP formulations, avoiding the inverse, become relatively faster.

Two versions of the genomic marker matrix encodings were tested. The first was VanRaden method 1 matrix (${\mathbf{Z}}_m^{vR1}$) where the marker information is centered around the mean of the observed genotyped animals, and scaled [15]. The second was “−1,0,1 encoding” (${\mathbf{Z}}_m^{-1,0,1}$) where the 0,1,2 genotypes were assigned values of −1,0,1, then scaled. In both cases, the centered ${\mathbf{Z}}_m$ was scaled by dividing by $\sqrt{\sum _{i=1}^{m} 2p_i (1-p_i)}$ where $m$ is the number of markers, and $p_i$ is the allele frequency of marker $i$. In case of −1,0,1 coding, the allele frequencies were all $p_i=0.5$, and the scaling factor was $\sqrt{\frac{m}{2}}$. In VanRaden 1, the allele frequencies were those of the observed genotypes.

## Results and discussion

### Efficiency

In general, all sparse matrices in this study were very sparse. Table 1 shows the sizes and sparsity of the various sparse matrices and their decomposition in the test case. The most important matrix is ${\mathbf{L}}_1$ which is used in the imputation process of Eq. (23). Compared to the matrix ${\mathbf{A}}^{11}$, to which the Cholesky matrix ${\mathbf{L}}_1$ belongs, there is only a minor additional fill-in. Both matrices have less than three non-zero elements on average on each row or column. Note that the number of non-zeros in ${\mathbf{A}}^{-1}$ equals the sum of those in ${\mathbf{A}}^{11}$, ${\mathbf{A}}^{22}$, and twice in ${\mathbf{A}}^{12}$. On average, matrix ${\mathbf{A}}^{12}$ has less than one non-zero element on a row, but almost 22 non-zero elements on a column. This matrix has elements due to non-genotyped animals being offspring, parents and/or mates to a genotyped animal.

**Table 1:** Number of rows ($N_r$), columns ($N_c$), non-zeros ($N_z$), and mean number of non-zeros on row or column ($M_z$) by matrix used in MME coefficient matrices in the test case with 73,579 animals

Matrix	$N_r$	$N_c$	$N_z$	$M_z$
${\mathbf{A}}^{-1}$	73,579	73,579	235,669^a^	3.20^a^
${\mathbf{A}}^{11}$	70,694	70,694	168,818^a^	2.39^a^
${\mathbf{A}}^{12}$	70,694	2885	62,489	0.88/21.66
${\mathbf{A}}^{22}$	2885	2885	4362^a^	1.51^a^
${\mathbf{L}}$	73,579	73,579	191,026	2.60
${\mathbf{L}}_1$	70,694	70,694	181,536	2.57
${\widehat{{\mathbf{A}}}}^{-1}$	6833	6833	22,612^a^	3.31^a^
$\widehat{{\mathbf{L}}}$	6833	6833	18,173	2.66

${\mathbf{L}}$ and ${\mathbf{L}}_1$ are Cholesky decomposition matrices of ${\mathbf{A}}^{-1}$ (inverse of pedigree relationship matrix) and ${\mathbf{A}}^{11}$ (submatrix of ${\mathbf{A}}^{-1}$), respectively. $\widehat{{\mathbf{A}}}$ and $\widehat{{\mathbf{L}}}$ are the corresponding matrices for smaller pedigree of genotyped animals and their ancestors

^a^Non-zeros of symmetric matrices counted from the lower/upper triangular region only

In this study, an implicit “on-the-fly” imputation process was used. Fernando et al. [6] suggested imputing the genotypes for the non-genotyped animals, i.e. computing the predicted values of genotypes based on pedigree. Their approach for Bayesian estimation of SNP effects requires, at least the diagonal elements of, the block that pertains to SNP effects:

$$\begin{aligned} {\mathbf{Z}}_{imp}^{\prime}{\mathbf{Z}}_1^{\prime}{{\mathbf{R}}}^{-1}{\mathbf{Z}}_1{\mathbf{Z}}_{imp} + {\mathbf{Z}}_m^{\prime}{\mathbf{Z}}_2^{\prime}{{\mathbf{R}}}^{-1}{\mathbf{Z}}_2{\mathbf{Z}}_m, \end{aligned}$$

in equivalent MME 2 or 3 (basic and expanded RPG ssSNP-BLUP) with $w = 0$ and where ${\mathbf{Z}}_{imp} = {\mathbf{A}}_{imp}{\mathbf{Z}}_m$ contains the imputed genotypes. If genotypes in ${\mathbf{Z}}_{imp}$ had been predicted for the non-genotyped animals in our data and stored using single precision accuracy, then about 11 gigabytes would have been read from the file or kept in memory. Note that the imputed genotypes for the non-genotyped animals are real numbers, so they cannot be stored as integers while retaining full accuracy. Also, the marker matrix is a dense matrix of size equal to the number of animals times markers. For this small example, an 11 GB file is quite a large extra file to be read when all the other files would take only some tens of megabytes. Our implicit on-the-fly implementation process, however, works with sparse matrices. For our example case, for the largest MME size, this means storing 372,562 non-zeros due to sparse matrices ${\mathbf{L}}$, and ${\mathbf{L}}_1$, i.e., about 3 megabytes using double precision.

PCG iteration was implemented such that all operations were matrix by vector products. Thus, there was no need to build matrix ${\mathbf{Z}}_{imp}^{\prime}{\mathbf{Z}}_1^{\prime}{{\mathbf{R}}}^{-1}{\mathbf{Z}}_1{\mathbf{Z}}_{imp}$ but instead the required computations were performed stepwise from right to left in ${\mathbf{Z}}_{imp}^{\prime}({\mathbf{Z}}_1^{\prime}({{\mathbf{R}}}^{-1}({\mathbf{Z}}_1({\mathbf{Z}}_{imp}{\mathbf{v}}))))$ where ${\mathbf{v}}$ is a vector. This approach saves memory and allows fast computations [19].

Because of the very sparse pedigree relationship (${{\mathbf{A}}}^{-1}$) and factorization (${\mathbf{L}}$) matrices, the operations on each PCG iteration step that involve inverses of the sparse matrices, e.g. the imputation operations, can be calculated in linear time with respect to the number of individuals. The matrix multiplication of the marker matrix ${\mathbf{Z}}_m$ is also linear if the number of markers is assumed to be constant. Hence, the cubic complexity of inverting the genomic relationship matrix can be replaced by linear computational complexity of ssSNP-BLUP formulations. This holds if the iteration counts remain low.

### Accuracy

All the derived ssGBLUP MME formulations in this paper were linearly equivalent. Consequently, the final iterative solutions by all formulations were numerically equal and dictated only by the convergence tolerance. All formulations had “real” relative residual errors $\widetilde{{\mathbf{e}}}_i$ in Eq. (66) of $10^{-10}$ scale when using the residual convergence tolerance $10^{-12}$ in pcg. Thus, all formulations indicated convergence to the same solutions, and no divergence of the iterative method was observed.

### Speed of iterative convergence

#### Genetic relationship: VanRaden 1

Without preconditioning, the original ssGBLUP achieved relative convergence of $10^{-12}$ in about 360 iterations when VanRaden 1 marker matrix encodings (${\mathbf{Z}}_{m}^{vR1}$) and assumed heritability 0.5 were used (Table 2). The iteration counts of the first three equivalent formulations 1 to 3 were higher than with the original ssGBLUP. In particular, the third formulation (40), i.e. expanded RPG ssSNP-BLUP, had clearly the worst iteration counts although the formulation is very similar to the second formulation (39), basic RPG ssSNP-BLUP. In the third formulation, the full ${\mathbf{A}}$ matrix was used instead of ${\mathbf{A}}_{22}$ matrix having the genotyped relationships. This increased the number of unknowns by the number of non-genotyped animals, in our case c. 64%.

**Table 2:** Number of iterations in PCG of linearly equivalent ssGBLUP MME using marker matrix ${\mathbf{Z}}_m^{vR1}$ (VanRaden 1), convergence tolerance $10^{-12}$, heritability 0.5, and no preconditioning under different polygenic proportions* w*

MME	Size	Weight ${w}$					
		0.00	0.01	0.10	0.20	0.30	1.00
Orig.	73,580	–	362	358	358	355	357
1	73,580	–	536	512	536	523	536
2	111,106	646	1203	1085	987	886	536
3	181,800	646	4023	3660	3307	3040	355
4	181,800	193	196	193	191	190	182
5	181,800	193	197	194	192	190	181
6	115,054	193	196	193	191	190	182

MME, original ssGBLUP; 1, basic equivalent ssGBLUP; 2, basic RPG ssSNP-BLUP; 3, expanded RPG ssSNP-BLUP; 4, orthogonal ssSNP-BLUP; 5, orthogonal expanded ssSNP-BLUP; 6, reduced orthogonal ssSNP-BLUP

When $w = 1$, all equivalent MME coincide with the traditional animal model with no genomic information and the numbers of iterations are smaller, even for the badly behaving third formulation (40). When $w = 0$, the original ssGBLUP and the first equivalent MME (36), basic equivalent ssGBLUP, cannot be calculated because of the singularity in the inverse of the genomic relationship matrix ${{\mathbf{G}}_g}^{-1}$ in the test case. The numbers of iterations of the second (39) and third (40) MME are much lower in this case, which implies that the higher iteration counts are due to the RPG terms.

All fully orthogonalized equivalent ssSNP-BLUP MME 4 to 6 needed about half the number of iterations of the original ssGBLUP in all cases of $w$. Note that the fifth formulation (60), i.e. orthogonal expanded ssSNP-BLUP, is an orthogonalized version of the badly behaving third formulation (40) while the fourth (59), i.e. orthogonal ssSNP-BLUP, and the sixth (65), i.e. reduced orthogonal ssSNP-BLUP, are orthogonalized versions of the second MME (39). The applied orthogonalization clearly reduced the number of iterations. Of the three orthogonalized ssSNP-BLUP formulations 4 to 6, the last, number 6 (65) is preferred because it has the smallest number of unknowns (115,054).

It should be noted that the convergence results apply only on the data used in the example. The equivalent MME formulation 2 (39), i.e. basic RPG ssSNP-BLUP, had 37,526 equations more than the MME formulation 1 (36), i.e. basic equivalent ssGBLUP. These extra equations were for the SNP solutions. Furthermore, equivalent MME 3 (40), i.e. expanded RPG ssSNP-BLUP, had again 70,694 new equations associated with RPG of non-genotyped animals. It remains to be tested whether MME 2 and 3 are more competitive when the number of genotyped animals is either close to or larger than the number of SNPs.

When diagonal preconditioning was applied, the original ssGBLUP achieved relative convergence of $10^{-12}$ much faster than without preconditioning, in about 60 iterations (vs. 360 iterations) (Table 3). Similarly, the first equivalent MME formulation gained from the preconditioning, whereas the convergence speed of the formulations 2 and 3 was about the same as without preconditioning.

**Table 3:** Number of iterations in PCG of linearly equivalent ssGBLUP MME using marker matrix ${\mathbf{Z}}_m^{vR1}$ (VanRaden 1), convergence tolerance $10^{-12}$, heritability 0.5, and diagonal preconditioning under different polygenic proportions *w*

MME	Size	Weight ${w}$					
		0.00	0.01	0.10	0.20	0.30	1.00
Orig.	73,580	–	93	62	59	57	57
1	73,580	–	152	153	158	158	171
2	111,106	847	1176	1099	1007	950	171
3	181,800	847	4058	3658	3195	2873	57
4	181,800	442	443	702	853	920	283
5	181,800	442	455	720	868	917	124
6	115,054	442	443	702	853	920	283

MME, original ssGBLUP; 1, basic equivalent ssGBLUP; 2, basic RPG ssSNP-BLUP; 3, expanded RPG ssSNP-BLUP; 4, orthogonal ssSNP-BLUP; 5, orthogonal expanded ssSNP-BLUP; 6, reduced orthogonal ssSNP-BLUP

However, all fully orthogonalized ssSNP-BLUP formulations 4 to 6 converged much more slowly with diagonal preconditioning. For these formulations, the inverse of the diagonal of the MME matrix is not a good approximation of the inverse MME coefficient matrix. This could mean that the inverse MME coefficient matrix is not diagonally dominant or that the off-diagonal parts of the MME matrix contribute to the diagonal of the inverse MME matrix.

The same dataset was analyzed using lower heritability values of 0.2 and 0.1 as well. This change in heritability had a large impact on convergence. Lower heritability leads to an increased relative weight on the variance matrix ${\mathbf{H}}^{-1}$ in MME of the original ssGBLUP. Consequently, the number of PCG iterations until convergence is expected to increase. However, in ssSNP-BLUP versions 4 to 6 the number of iterations decreased when heritability decreased. For these, the relative weight multiplies the orthogonalized variance matrix, i.e. the identity matrix, which grows dominant. In a single trait case

67 $$\begin{aligned} \widetilde{{\mathbf{Z}}}_i^{\prime}\widetilde{{\mathbf{Z}}}_i + \lambda {\mathbf{I}}\xrightarrow [h^2 \rightarrow 0]{} \lambda {\mathbf{I}}, \end{aligned}$$

because $\lambda$ increases when heritability $h^2$ is small:

68 $$\begin{aligned} \lambda = \frac{1-h^2}{h^2} \xrightarrow [h^2 \rightarrow 0]{} \infty . \end{aligned}$$

With a lower heritability of 0.1, the orthogonalized equivalent ssSNP-BLUP formulations 4 to 6 achieved a relative convergence of $10^{-12}$ more quickly, in about 70 iterations without preconditioning (Table 4) whereas the original ssGBLUP needed 130 iterations with diagonal preconditioning (Table 5). Equivalent formulations 1 to 3 gained from the diagonal preconditioning but the orthogonalized formulations 4 to 6, again, did not.

**Table 4:** Number of iterations in PCG of linearly equivalent ssGBLUP MME using marker matrix ${\mathbf{Z}}_m^{vR1}$ (VanRaden 1), convergence tolerance $10^{-12}$, heritability 0.1, and no preconditioning under different polygenic proportions *w*

MME	Size	Weight ${w}$					
		0.00	0.01	0.10	0.20	0.30	1.00
Orig.	73,580	–	619	622	624	624	623
1	73,580	–	758	733	701	728	744
2	111,106	736	1005	970	907	836	748
3	181,800	736	3422	3315	3154	2934	623
4	181,800	74	74	72	72	71	71
5	181,800	74	73	72	70	71	70
6	115,054	74	74	72	72	71	71

MME, original ssGBLUP; 1, basic equivalent ssGBLUP; 2, basic RPG ssSNP-BLUP; 3, expanded RPG ssSNP-BLUP; 4, orthogonal ssSNP-BLUP; 5, orthogonal expanded ssSNP-BLUP; 6, reduced orthogonal ssSNP-BLUP

**Table 5:** Number of iterations in PCG of linearly equivalent ssGBLUP MME using marker matrix ${\mathbf{Z}}_m^{vR1}$ (VanRaden 1), convergence tolerance $10^{-12}$, heritability 0.1, and diagonal preconditioning under different polygenic proportions *w*

MME	Size	Weight ${w}$					
		0.00	0.01	0.10	0.20	0.30	1.00
Orig.	73,580	–	178	131	126	122	119
1	73,580	–	191	156	148	145	139
2	111,106	438	491	459	429	398	139
3	181,800	438	1732	1592	1416	1247	119
4	181,800	107	105	130	151	160	82
5	181,800	107	107	132	151	162	49
6	115,054	107	105	130	151	160	82

MME, original ssGBLUP; 1, basic equivalent ssGBLUP; 2, basic RPG ssSNP-BLUP; 3, expanded RPG ssSNP-BLUP; 4, orthogonal ssSNP-BLUP; 5, orthogonal expanded ssSNP-BLUP; 6, reduced orthogonal ssSNP-BLUP

When the heritability was equal to 0.2, the diagonally preconditioned original ssGBLUP and non-preconditioned orthogonalized ssSNP-BLUP formulations all achieved relative convergence of $10^{-12}$ in about the same 100 iterations (results not shown).

#### Genetic relationship: −1,0,1 encoding

Tables 6 and 7 show the numbers of pcg iterations needed for convergence using marker matrix ${\mathbf{Z}}_m^{-1,0,1}$ (−1,0,1 encoding) and a heritability of 0.5, without and with diagonal preconditioning, respectively. The results were similar to the ${\mathbf{Z}}_m^{vR1}$ (VanRaden 1) case (Tables 2, 3) but the number of required iterations was overall a little smaller, at least in the preconditioned case. In the pure SNP-BLUP type of computations, it is expected that observed genotype centering will lead to faster convergence, at least in Gibbs sampling or Gauss–Seidel type of iterations [23]. One reason for the poorer convergence may be that base population allele frequencies were not used in VanRaden 1 as advocated in [15]. Use of base population allele frequencies might give a genomic relationship matrix that is more appropriate in ssGBLUP, and deviations from this matrix may lead to poorer convergence.

**Table 6:** Number of iterations in PCG of linearly equivalent MME using marker matrix ${\mathbf{Z}}_m^{-1,0,1}$ (−1,0,1 encoding), convergence tolerance $10^{-12}$, heritability 0.5, and no preconditioning under different polygenic proportions *w*

MME	Size	Weight ${w}$					
		0.00	0.01	0.10	0.20	0.30	1.00
Orig.	73,580	–	372	366	362	364	357
1	73,580	–	518	512	512	515	536
2	111,106	620	1141	1082	996	896	536
3	181,800	620	3794	3586	3390	3096	355
4	181,800	189	193	192	190	190	182
5	181,800	189	193	191	190	192	181
6	115,054	189	193	192	190	190	182

MME, original ssGBLUP; 1, basic equivalent ssGBLUP; 2, basic RPG ssSNP-BLUP; 3, expanded RPG ssSNP-BLUP; 4, orthogonal ssSNP-BLUP; 5, orthogonal expanded ssSNP-BLUP; 6, reduced orthogonal ssSNP-BLUP

**Table 7:** Number of iterations in PCG of linearly equivalent MME using marker matrix ${\mathbf{Z}}_m^{-1,0,1}$ (−1,0,1 encoding), convergence tolerance $10^{-12}$, heritability 0.5, and diagonal preconditioning under different polygenic proportions *w*

MME	Size	Weight ${w}$					
		0.00	0.01	0.10	0.20	0.30	1.00
Orig.	73,580	–	96	64	60	58	57
1	73,580	–	144	144	147	152	171
2	111,106	785	1059	1068	1010	953	171
3	181,800	785	3570	3411	3080	2826	57
4	181,800	418	413	636	762	817	283
5	181,800	418	423	673	805	857	124
6	115,054	418	413	636	762	817	283

MME, original ssGBLUP; 1, basic equivalent ssGBLUP; 2, basic RPG ssSNP-BLUP; 3, expanded RPG ssSNP-BLUP; 4, orthogonal ssSNP-BLUP; 5, orthogonal expanded ssSNP-BLUP; 6, reduced orthogonal ssSNP-BLUP

### Compatibility of the genomic relationship matrix ${\mathbf{G}}_w$

Convergence properties of the PCG method depend on the model used, data, and parameters. The model in our statistical analysis was very simple but we used different genomic relationship matrices. When genomic data is involved, several approaches are available to construct the genomic relationship matrix and the marker matrix ${\mathbf{Z}}_m$. Previously, Strandén and Christensen [23] had shown that differences in ${\mathbf{Z}}_m$ marker matrix in SNP-BLUP type models can give different mixing properties in Markov chain Monte Carlo computations. In ssGBLUP, the genomic and pedigree relationship matrices need to be constructed properly in order to avoid bias in the breeding values. We used different genomic relationship matrices by changing the $w$ parameter without trying to maximize prediction ability in our data. Our choice for the family of genomic relationship matrices showed differences in convergence that may be partly due to, a potentially suboptimal, ${{\mathbf{H}}}^{-1}$ matrix.

In practice, the genomic relationship matrix in ssGBLUP or ssSNP-BLUP can be built such that it is “compatible” with the pedigree relationship matrix. There are several strategies for this e.g., [24, 25]. For each strategy an equivalent ssSNP-BLUP can be derived similarly as done for the family of relationship matrices in our study. For example, when the genomic relationship matrix has the form $a {\mathbf{1}}{\mathbf{1}}^{\prime} + {\mathbf{G}}_g$ instead of ${\mathbf{G}}_w$, there will be one effect due to $a {\mathbf{1}}{\mathbf{1}}^{\prime}$ instead of the residual polygenic effects. The equations for the marker effects remain the same.

Some studies have indicated that the ${({\mathbf{A}}_{22})}^{-1}$ matrix in ssGBLUP should be scaled by a factor less than 1, e.g., [26, 27]. This suggests that not only the genomic relationship matrix should be carefully constructed but also the pedigree relationship matrix should be adjusted. This has been properly formulated in the metafounders approach [28] where the pedigree relationship matrix is modified.

### Comparison to other approaches

The model by Fernando et al. [6] did not include RPG effects. The existence of RPG effects can be justified because not all the additive genetic variation can be explained by marker genotypes [2, 29]. Moreover Goddard et al. [30] suggested that with a finite number of markers, estimates of the genomic relationships are subject to error. The error can be due to sampling variation or inaccuracies in the analysis, and is inversely related to the number of markers in the analysis. In practice, the RPG has been included in most genomic evaluations because a moderate $w$ is known to reduce the prediction bias in young selection candidates [27]. However, $w$ might not have notable effects in single-step models [27].

The RPG effect could be included in the models of Fernando et al. [6] as a general random effect with $Var({\mathbf{u}}) \sim N({\mathbf{0}}, w {\mathbf{A}})$. However, when the same observations of non-genotyped animals in [6] are modelled by RPG, the iterative approaches might face problems for separating the RPG and the “imputation residual” [6] (i.e. ${\mathbf{a}}_1$) effects from each other. This was clearly visible in our equivalent MME 3 (40), i.e. expanded RPG ssSNP-BLUP, which showed very poor convergence.

When RPG was not included ($w = 0$) in equivalent MME 2 and 3, basic and expanded RPG ssSNP-BLUP, they converged much faster. However, all the other equivalent MME alternatives reached convergence with a smaller number of iterations.

Liu et al. [8] proposed a different approach for the use of marker effects in single-step models. Their model for the observations did not include the SNP effects, but instead the SNP effects were introduced into the MME as augmented effects correlated with the aggregated genomic breeding values, i.e. the sum of the genomic breeding value and the RPG effect. In this way, the marker matrix generated the dependencies among genotyped animals similar to genomic relationships in the ${\mathbf{H}}$ matrix. However, the MME by Liu et al. [8] was reported to have problematic convergence properties [8] (Liu Z: personal communication). This may have been due to poor condition number of MME coefficient matrix which resulted from large off-diagonal elements in blocks connecting marker effects and aggregated genomic values.

## Conclusions

A procedure was presented to derive linearly equivalent MME formulations for ssGBLUP. Six ssGBLUP based MME were derived, of which five were ssSNP-BLUP. In ssSNP-BLUP, inversion of the genomic relationship matrix is avoided. Three of the derived formulations were fully orthogonalized such that all random effects had diagonal covariance matrices.

All matrix operations and matrices, except the marker matrix ${\mathbf{Z}}_m$, were expressed using sparse matrices and sparse decompositions. During the iteration, the on-the-fly imputation from the genotyped to the non-genotyped animals was used on the genomic breeding values and residual polygenic effects without any need to explicitly predict and store genotypes of relatives of genotyped animals. All implementations used efficient matrix times vector operations where very sparse matrices were saved in memory. This enables efficient iteration on data-based implementations for large datasets and models.

All the derived MME gave exactly the same breeding value estimates at convergence with the small test data. Using the expected heritability of 0.5, the fully orthogonalized non-preconditioned ssSNP-BLUP formulations needed more iterations than the diagonally preconditioned original ssGBLUP. However, the number of iterations depended on the heritability value used. With a smaller heritability (0.1), the non-preconditioned ssSNP-BLUP formulations needed less iterations. The ssSNP-BLUP formulations did not benefit from diagonal preconditioner in PCG iteration.

The blending of the genomic relationship matrix ${\mathbf{G}}_g$ with the pedigree relationship matrix ${\mathbf{A}}_{22}$ was transformed into an additional residual polygenic effect in MME. The number of estimated additional random effects from all animals was reduced to include genotyped and their non-genotyped ancestors only. However, this did not improve convergence, although the number of unknowns to solve was smaller. When no residual polygenic effect was included, the ssSNP-BLUP models converged with a smaller number of iterations.

In conclusion, inversion of the large and dense genomic relationship matrix in the ssGBLUP can be avoided by using fully orthogonalized ssSNP-BLUP formulations. Although the new algorithms are more complicated than the original ssGBLUP, numerical efficiency is better when the number of genotyped individuals is large. The number of iterations until convergence by PCG was smaller in orthogonalized ssSNP-BLUP than in the original ssGBLUP when the heritability was low, but increased above that of the original ssGBLUP when heritability was higher.

The performance of the new MME should be further tested by analyzing data with more genotyped and non-genotyped animals.
